# A community-based prospective cohort study of dengue viral infection in Malaysia: the study protocol

**DOI:** 10.1186/s40249-016-0172-3

**Published:** 2016-08-11

**Authors:** Nowrozy Kamar Jahan, Mohtar Pungut Ahmad, Amreeta Dhanoa, Cheong Yuet Meng, Lau Wee Ming, Daniel D. Reidpath, Pascale Allotey, Anuar Zaini, Maude Elvira Phipps, Quek Kia Fatt, Aman Bin Rabu, Rowther Sirajudeen, Ahmad AbdulBasitz Ahmad Fatan, Faidzal Adlee Ghafar, Hamdan Bin Ahmad, Iekhsan Othman, Sharifah SyedHassan

**Affiliations:** 1 Jeffrey Cheah School of Medicine and Health Sciences, Monash University Malaysia, Jalan Lagoon Selatan, Bandar Sunway, Petaling Jaya, 47500 Malaysia; 2 Segamat District Public Health Office, Ministry of Health Malaysia, Peti Surat 102, Jalan Gudang Ubat, Kampung Gubah, Segamat, Johor Darul Takzim 85000 Malaysia; 3 Infection and Immunity Cluster, Tropical Medicine and Biology Platform, Monash University Malaysia, Jalan Lagoon Selatan, Bandar Sunway, Petaling Jaya, 47500 Malaysia; 4 South East Asia Community Observatory (SEACO), 146 Jalan Sia Her Yam, Suite 601-606, Wisma Centrepoint, Segamat, Johor Darul Takzim 85000 Malaysia; 5 Hospital Segamat, Ministry of Health Malaysia , KM 6, Jalan Genuang, Segamat, Johor Darul Takzim 85000 Malaysia; 6 University Sains Malaysia, Gelugor, Penang 11800 Malaysia

**Keywords:** Dengue, Community-based, Prospective cohort study, Health and demographic surveillance site, SEACO, Protocol

## Abstract

**Background:**

Globally, dengue infections constitute a significant public health burden. In recent decades, Malaysia has become a dengue hyper-endemic country with the co-circulation of the four dengue virus serotypes. The cyclical dominance of sub-types contributes to a pattern of major outbreaks. The consequences can be observed in the rising incidence of reported dengue cases and dengue related deaths. Understanding the complex interaction of the dengue virus, its human hosts and the mosquito vectors at the community level may help develop strategies for addressing the problem.

**Methods:**

A prospective cohort study will be conducted in Segamat district of Johor State in Peninsular Malaysia. Researchers received approval from the Malaysian Medical Research Ethics Committee and Monash University Human Research Ethics Committee. The study will be conducted at a Malaysian based health and demographic surveillance site over a 1 year period in three different settings (urban, semi-urban and rural). The study will recruit healthy adults (male and female) aged 18 years and over, from three ethnic groups (Malay, Chinese and Indian). The sample size calculated using the Fleiss method with continuity correction is 333. Sero-surveillance of participants will be undertaken to identify asymptomatic, otherwise healthy cases; cases with dengue fever who are managed as out-patients; and cases with dengue fever admitted to a hospital. A genetic analysis of the participants will be undertaken to determine whether there is a relationship between genetic predisposition and disease severity. A detailed medical history, past history of dengue infection, vaccination history against other flaviviruses such as Japanese encephalitis and Yellow fever, and the family history of dengue infection will also be collected. In addition, a mosquito surveillance will be carried out simultaneously in recruitment areas to determine the molecular taxonomy of circulating vectors.

**Discussion:**

The research findings will estimate the burden of asymptomatic and symptomatic dengue at the community level. It will also examine the relationship between virus serotypes and host genotypes, and the association of the clinical manifestation of the early phase with the entire course of illness.

**Electronic supplementary material:**

The online version of this article (doi:10.1186/s40249-016-0172-3) contains supplementary material, which is available to authorized users.

## Multilingual abstracts

Please see Additional file 1 for translations of the abstract into the six official working languages of the United Nations.

## Background

Globally, dengue infections constitute a significant public health burden. Dengue infections account for approximately 1.14 million disability-adjusted life-years (DALYs), and 576 900 years of life lost due to premature death [1]. In 2010, 294 million asymptomatic and 96 million symptomatic dengue infections were estimated to have occurred worldwide, with an asymptomatic to symptomatic infection ratio of almost 3 to 1. The greatest estimated burden of dengue is found in the Asian region [2]. Nearly two-thirds of the global population is at risk, predominantly in the Asia-Pacific region [3]. Dengue is endemic in the Southeast Asian tropical countries where rapid urbanization has resulted in an increased density of susceptible human hosts for the dengue virus. Also, water-holding containers of the construction industry promotes the geographical expansion of the vector by increasing mosquito breeding sites in urban areas [4]. Dengue is, furthermore, a significant economic burden of the region [5].

Dengue is caused by a virus belonging to the *Flaviviridae* family. The dengue virus (DENV) has four antigenically related, but immunologically distinct serotypes (DENV-1, DENV-2, DENV-3, and DENV-4). The virus is hyper-endemic in Malaysia; and the four DENV serotypes have been co-circulating for the last two decades. There is a pattern of cyclical dominance of sub-types contributing to the development of frequent major dengue epidemics [6, 7]. The virus is mainly transmitted by infected female *Aedes aegypti* mosquitoes. In Malaysia, over the last few decades, this mosquito-borne virus has been spreading rapidly. In 1973, Malaysia experienced its first major outbreak [8] and since then, the incidence has sharply increased. From 2000 to 2010, the number of symptomatic dengue cases increased approximately sevenfold (7 103 in 2000 to 46 171 in 2010); during the same period, a shift in the age distribution from children towards adults was also observable [9]. Although there is a variable trend of annual dengue related deaths; these have increased threefold from 45 deaths in 2000 to 134 deaths in 2010 [10]. The numbers continue to rise: 103 610 symptomatic dengue cases and 199 deaths were reported in 2014 [11], and 111 285 symptomatic cases and 301 deaths in 2015. The incidence of dengue cases was higher in Malaysia than that of the neighbouring countries (e.g. Singapore 10 470 cases, Viet Nam 79 912 cases, Cambodia 15 015 cases, Lao PDR 1 912 cases ) except Philippines (169 435 cases) [12]. In Thailand, 140 000 dengue cases were recorded at the same time period [13].

Empirical studies suggest that the primary infection induces a lifelong protective immunity against the homologous DENV serotype, but confers only partial and transient protection against subsequent infection by the other three heterologous DENV serotypes. In addition, secondary infection by heterologous DENV serotypes is usually associated with the clinical manifestations of severe dengue. This mainly occurs due to an immune enhancement mechanism whereby preexisting antibodies fail to counteract the heterologous virus; as a result the virus replicates to amplify the infection [14–16].

According to the World Health Organization (WHO), a person can develop a symptomatic dengue infection with or without warning signs [17], and it represents only a small fraction of the overall disease burden. Further evidence [18] suggests that less than 10 % of dengue infections develop clinical manifestations, while the remaining 90 % of DENV infections are sub-clinical. The sub-clinical host then develops either no clinical manifestation or a mild illness which often goes unrecognised and unreported. The sub-clinical host also becomes a potential target for heterologous DENV serotype infection with the clinical complications associated with that.

Resistance, susceptibility or the severity of the infection depends not only on the virulence of virus, but also on the host’s genetic factors, especially the presence of human leukocyte antigen (HLA) on chromosome 6, T lymphocytes, antibody receptors, immune mediators, and cytokines which regulate the immune-effects [19–22]. Activation of memory dengue virus-specific T lymphocytes and monocytes induces the production of cytokines and other chemical immune mediators which increases capillary permeability leading to severe dengue, i.e. extensive plasma leakage resulting in shock [23–25]. Harapan et al. [26] found an association between different HLAs (Class I and Class II) and dengue infection vulnerability, protection and severity. A large case-control study in Vietnam [27] identified the impact of non-HLA host genetic factors especially on the susceptibility of dengue haemorrhagic fever. The Malaysian genetic study found a relationship between a host’s ethnicity and susceptibility to and/or defence against DENV infection [28].

Unlike cross-sectional studies, prospective cohort studies can provide insights into the dynamics of DENV transmission in a population, allowing the exploration of the diversity of DENV serotype-specific transmission and the impact of host factors and pre-existing dengue antibodies on dengue disease severity [29, 30]. A recent systematic review of dengue incidence and death outcomes found that most of the data came from cross-sectional studies at the national and local level [9]. Moreover, other research conducted in Malaysia, was found to rely on retrospective and hospital-based designs; few prospective studies have been conducted on adult and pediatric patients [6]. These earlier studies offered a limited understanding of the complex interaction between the dengue virus, human hosts and mosquito vectors.

To generate comprehensive evidence about the full burden of dengue infection at the community level in Malaysia, it is important to follow a healthy cohort over time. During the period of surveillance, participants may remain healthy, some may become infected but asymptomatic, others may become infected and symptomatic. These data provide an estimate of the ratio of asymptomatic (sub-clinical) and symptomatic (clinical) cases, as well as the ratio of uninfected and infected cases. The study will also allow investigations of a potential reservoir of primary and secondary infections by different DENV serotypes at the community level, the human hosts’ genetic profiles, current and previous immunological responses, the socio-economic and demographic factors, and vector factors in relation to the circulating DENV serotypes.

In this manuscript, we outline the protocol for a prospective community-based cohort study. This study aims to determine several specific objectives: the spectrum of DENV infection at the community level; the association of primary and secondary antibody responses with the spectrum of disease severity; the relationship of DENV serotypes and relevant human host genotypes with disease severity; the impact of socio-economic and demographic factors in dengue infection; and the molecular taxonomy of the circulating mosquito vectors.

## Methods

### Study site

The prospective cohort study will be based in the district of Segamat in the state of Johor in Peninsular Malaysia. According to the latest national census in 2010, the total population of the 11 sub-districts of Segamat district was 183 341 [31]. More specifically, the study will be conducted within the South East Asia Community Observatory (SEACO), a Malaysian based health and demographic surveillance site (HDSS) established in November 2011, which operates in five of the 11 sub-districts of Segamat (Sungai Segamat, Bekok, Chaah, Gemereh and Jabi). In collaboration with national and international researchers, SEACO’s trained field research team conducts the multi-disciplinary, community-based, health related research [32]. During the initial census establishing SEACO, 38 228 respondents joined, representing an 81 % response rate. From the population pyramid of SEACO respondents, it was found that 83 % of Malaysian citizens are 20 years of age and above [33].

In Segamat, the number of reported dengue cases has steadily increased over the last 3 years (74 cases in 2013, 190 cases in 2014 and 284 cases in 2015). In 2013, Johor and the adjacent state of Malacca had the highest dengue case fatality rates in Malaysia. Johor also bears the second greatest burden of dengue cases (11 %) in Malaysia [7].

The study will be conducted over a year across the three settings (urban, semi-urban and rural) in one of the SEACO subdistricts (Sungai Segamat). The subdistrict was chosen because, based on data from the Segamat District Public Health Office, its urban area has been the epicenter of dengue cases since 2009. In 2014, dengue cases were also reported in the adjacent semi-urban areas. This suggests that these areas may have latent reservoirs of asymptomatic dengue infections. Although no dengue cases were reported in the adjacent rural area, it was included based on the estimated reach of the dengue mosquito population [34, 35].

### Study population

Due to the shifting demographic, towards an increasingly adult population [9], the study will recruit healthy adults (male and female) aged 18 years and over, from three ethnic groups (Malay, Chinese and Indian) living within the subdistrict of Sungai Segamat who are enroled in the SEACO HDSS. Engaging the SEACO respondents as a potential study population is advantageous because their basic socio-economic and demographic information is available in the SEACO database, and they have already shown a preparedness to participate in longitudinal health research. For the purposes of this study, therefore, data collection will be restricted to dengue specific data and it will be linked to the respondents’ socioeconomic and demographic data.

Potential participants who suffer from any fever on the day of enrolment will be excluded from the study as it aims to follow a healthy cohort estimating the ratio of uninfected and infected. The respondents who are suffering from any chronic illness or may not be physically capable of attending the blood collection centers will also be excluded. Pregnant women, who may face difficulties attending all four waves of data collection, will not be encouraged to join. Respondents with any bleeding disorder (e.g. Haemophilia) will also be excluded as it may increase the difficulty of diagnosing dengue hemorrhagic fever.

Each participant will receive a research specific identification card with a unique identification number. This unique number will be used as an identifier throughout the study period and will make the link between research specific information and blood samples which will be collected at the community and at the health facility levels. In the laboratory, the same unique identification number will be used to create barcodes for the test tubes. Two specific digits will be used at the end of each unique identification number to specify the waves and location of data collection. For example, at the community-based 1st wave, blood will be collected in two test tubes; the last two digits will be 01 and 02. If the participants remain healthy and do not suffer from dengue infection for 1 year, they will join the 2nd, 3rd and 4th waves of blood collection as a part of a sero-prevalence surveillance. In these cases, the last two digits will be 22, 33, and 44 respectively. If a participant suffers from a dengue infection and is treated as an out-patient or in-patient, the last two digits of the barcode will be 03 and 04 respectively.

### Estimated sample size

The sample size of 333 was calculated using the Fleiss formula with continuity correction [36]. The sample size was calculated based on the following information

i. The dengue incidence in the high risk area in Segamat (Exposed) = 5 %
ii. The dengue incidence in the low risk area in Segamat (Unexposed) = 1 %
iii. Ratio of sample size, Unexposed/Exposed = 1
iv. Two-sided significance level (1- α) = 95 %
v. Power of the study (1-β, % of chance of detecting) = 80 %
vi. α = 5 %
vii. Percent of Unexposed with Outcome = 1
viii. Percent of Exposed with Outcome = 5
ix. Odds Ratio = 5.2
x. Risk/Prevalence Ratio = 5
xi. Risk/Prevalence difference = 4

### Stakeholders engagement

Stakeholder engagement was integral to the development of the proposal and the basis for the collaboration between the District Public Health Office of the Malaysian Ministry of Health, the School of Medicine and Health Sciences in Monash University Malaysia and SEACO. Further support was obtained from the Segamat District Office and the community leaders.

As a part of the community engagement strategy, Community Engagement Committees (CEC) are formally constituted in each of the SEACO sub-districts [32]. Before commencing the study, all the active members of the CEC of Sungai Segamat sub-district will be briefed about the purpose of the research to obtain their suggestions for optimising participation.

In Malaysia, both public and private health care services are available. The Ministry of Health (MOH) provides health care service through its health clinics and hospitals [37]. In the Sungai Segamat sub-district, there are two public health clinics: Klinik Kesihatan (KK) Segamat and Klinik Kesihatan (KK) Bandar Putra and both health facilities will participate in this study. Each health clinic provides service to a population between 15 000 and 20 000. General practitioners (GPs) provide private health care services. The preference for attending specific general practitioners will be collected from the participants during enrolment. These preferred general practitioners will be invited to join the research. There is only one hospital in Segamat (Hospital Segamat), which is a 314 bed government hospital.

A network will be developed among the local health professionals who are working in the medical and emergency departments of Hospital Segamat and two health clinics (KK Segamat and KK Bandar Putra), and respondents’ preferred general practitioners with the research team. These health professionals will manage the study populations if they develop any clinical manifestations of dengue infection over the duration of the study period. In addition, the health professionals will collect research specific information and blood samples from the participants with dengue fever. SEACO research staff will be notified by the health professionals over the telephone if participants report with dengue fever.

#### Different cohorts of surveillance

To capture the full burden of dengue infection, sero-surveillance will be undertaken in three groups: healthy participants to identify asymptomatic cases; participants who develop dengue fever managed as out-patients, and participants who develop dengue fever admitted to hospital. In addition, a mosquito surveillance will be carried out at the same time that the community blood collection occurs.

- Sero-prevalence surveillance of healthy participants:

The sero-prevalence surveillance will be conducted on the recruited healthy study population. At the initial stage, they will receive an invitation to join the first wave at a local community hall. The first wave will also be used to collect baseline questionnaire data. To ensure that potential participants do not have a fever at enrolment, forehead temperature will be checked using an infrared forehead thermometer. Participants with a temperature of 37.5° centigrade or above will be excluded, and advised to visit a doctor for further management and treatment.

Nine millilitres (mls) of blood will be drawn by trained phlebotomists from the healthy study population. Five mls of the blood will be collected in a plain test tube with clot activator and gel separator, and will be later tested for dengue specific Immunoglobulin M (IgM) and ImmunoglobulinG (IgG) antibodies using PanBio (Australia) Dengue IgM and IgG Capture Enzyme-linked Immunosorbent Assay (ELISA) kits. In case of serum samples which are positive for IgM/IgG, a Plaque Reduction Neutralization Test (PRNT) will be carried out to identify the virus serotype/s. The antibody titre will be determined using an immunoperoxidase assay or serum neutralization/PRNT against each of the four DENV serotypes. In this way, human antibodies to dengue virus will be identified and quantified in healthy participants to detect whether they suffered from any asymptomatic recent dengue viral infection, and had any past infection.

Host genetic analyses will be done for Janus Kinase 1 (JAK1) and HLA with the remaining four mls of blood. This sample will be collected in an Ethylenediaminetetraacetic acid (EDTA) test tube and will be mixed with DNAgard. With new sequencing technologies that are able to perform long reads such as Pac Bio systems, more comprehensive HLA Class I & II haplotyping will be undertaken. Genotyping single nucleotide polymorphisms (SNPs) in JAK1 and Fc-gamma receptor (FcγR) will be followed by Bioinformatics analyses.

A detailed medical history, past history of dengue infection and other arboviral infections (e.g., Chikungunya, Zika), vaccination history against other flaviviruses such as Japanese encephalitis and Yellow fever, and the family history of dengue infection will also be collected from each enroled participant through a structured questionnaire in Open Data Kit (ODK) using an Android mobile device where error and verification checking will be embedded. It will ensure the quality of data collection, and allow quick and secure data uploading to the server. All the information will be considered as the baseline information.

Participants will be followed up once every 3 months for 1 year. During each follow up visit, five mls of blood will be collected in a plain test tube with clot activator and gel separator. The blood samples will be tested for dengue specific IgM and IgG antibodies to identify asymptomatic cases. In total, blood will be collected four times in 1 year: at baseline and on three additional occasions. A digital thermometer will be given as a token of appreciation and also serve for the patient to monitor any fever that occurs over the follow-up period. They will also receive a refrigerator magnet with a laminated information sheet with about what to do when they develop a fever. Participants will also receive fortnightly reminders via short message service (SMS). To monitor compliance with the study protocol, the SEACO field team will follow up the respondents over telephone and through home visits.

Collected blood will be stored appropriately according to a standard protocol. Blood in a plain test tube for serological tests will be kept at room temperature for at least 30–40 min to allow the blood to clot. The clotted whole blood with separated serum will be kept in an ice box to maintain a temperature of 4–8° centigrade. Clotted blood will be transported in an ice box from the blood collection hall to the laboratory in Segamat where it will be centrifuged and stored immediately in a refrigerator. An extra ice box will be available to create redundancy in the cold chain in case of an emergency. DNAgard mixed blood in an EDTA test tube will be stored at room temperature. Proper handling will be ensured during the transfer of the collected blood samples from the blood collection hall to the laboratory in Segamat, and finally to the research laboratory of Monash University Malaysia’s Sunway campus. Once a participant is confirmed to have dengue infection, they will be excluded from further participation in the sero-prevalence surveillance study.

b) Sero-surveillance of participants with dengue fever managed as out-patients:

Clinical manifestation of dengue infection may occur at any time and may remain unreported when the fever is mild, and participants may not feel that a visit to a health care provider is necessary. Therefore, the study population will be actively monitored through home visit and over the telephone. After the baseline blood collection, participants will be asked to monitor their body temperature during the 1-year study period. If they feel feverish, they will check their axillary temperature twice within 24 h. Sick participants will visit any of the selected health professionals of the network involved in this research when their axillary temperature is 37° centigrade or above.

In Malaysia, health care facilities have different working hours. Public health clinics and general practitioners mainly work during weekdays from 8 am to 4 pm. During weekdays from 8 am to 4 pm, the participants with a fever will visit either KK Segamat or KK Bandar Putra or any of the selected general practitioners, and during weekdays after 4 pm, weekends and public holidays, they are directed to visit the Accident and Emergency (A&E) Department of the Hospital Segamat, within 24 to 48 h of having a fever. While visiting the selected health professionals, the sick participants will show their research specific identification card to confirm their participation in the study, and trigger data collection procedures at the health facility.

The doctor-in-charge will re-check and record the temperature of the participants. All the health professionals involved in this study will be provided with rapid dengue diagnostic test kits: SD BIOLINE DengueDuo kits (Taiwan) for detection of nonstructural protein 1 (NS1). They will draw five mls of whole blood, perform the rapid diagnostic test, and store residual blood in a refrigerator. These blood samples will be collected by the research team on the same day and will be sent to the research laboratory for virus isolation and serotyping on the following day. The doctors will also complete a questionnaire to collect information on symptoms, clinical signs, the presence or absence of warning signs, provisional diagnosis and outcome (whether the patient is managed as non-dengue or dengue case).

If the result of NS1 rapid dengue diagnostic test is negative, the doctor will manage the patient as a non-dengue case, and will advise the patient to return to the clinic after 3 days to repeat the test. Doctors will also advise the patient to report back immediately if he or she develops any of the dengue warning signs (abdominal pain or tenderness, persistent vomiting, clinical fluid accumulation like pleural effusion/ascites, mucosal bleed, restlessness or lethargy, tender enlarged liver) over the next 3 days. They will refer the patient with a research specific referral slip to the A&E Department of Hospital Segamat for admission.

If the result of a rapid test is positive, the doctors will assess the clinical status of the patient. The patient will be managed as an outpatient if the patient is clinically well, stable and has no warning signs. They will be asked to return for follow-up. According to the MOH follow-up guideline, the patient will be monitored daily for haematocrit (HCT) and platelet counts. They will refer the patient to the A&E Department of Hospital Segamat for admission if the patient develops any warning signs, or has a laboratory result indicating increased HCT concurrent with a rapid decrease in the platelet count during the follow up period.

If the participants visit the A&E department of Hospital Segamat, and if NS1 rapid dengue diagnostic test is negative, the doctor-in-charge will advise the patient to visit either a KK or the GP after 3 days to repeat the Combo test. If the rapid diagnostic test is positive, they will provide a “follow up slip” and ask the patient to visit either a KK or the GP for daily follow up. In this way, the relationship between sero-prevalence and clinical manifestation will be assessed.

c) Sero-surveillance of participants with dengue fever admitted to hospital:

It is important to determine the variability of the spectrum of dengue infection, given the warning signs and factors involved in its clinical manifestations. Therefore, an ongoing surveillance of hospitalized participants will also be done. The patients will be admitted to Hospital Segamat if there are warning signs or laboratory results. After admission, the doctors of the Medical Department will manage the patient according to the National Clinical Guidelines. In addition, they will complete the research specific questionnaire, and will, on two occasions, draw five mls of blood for virus isolation and serotyping (1st draw will be on admission and 2nd draw will be 4–6 days later if the patient stays longer or, alternatively, at discharge). Blood samples will be stored in a refrigerator and will be collected by the research team on the same day before sending to the research laboratory for virus isolation and serotyping on the following day. They will also perform a rapid diagnostic test using the NS1 Combo test kit, if it was not done before admission. During discharge, they will ask the patients to visit either a KK or GP for follow up on designated dates. Follow up will be done after 1, 3, 6 and 12 months after discharge from the hospital. On each follow up, five mls of blood will be drawn for the IgM/IgG titration and Serotyping.

#### Mosquito surveillance

At the same time as the community blood collection is occurring in the healthy study population for the sero-prevalence surveillance, mosquito samples will also be collected for species identification, from the same three study sites. Molecular taxonomic studies on the mosquitoes will be conducted. The mosquitoes will be subjected to virus isolations in C636 and Vero cell lines, and viral serotypes will be identified by polymerase chain reaction (PCR) and serology. Next Generation Sequencing (NGS) will be performed on all virus isolates and their evolution. The relationship with Malaysian and other DENVs worldwide in the Gene Bank will be determined by phylogenetic analysis.

#### Storage of bio-specimens and research specific data

Bio-specimens will be stored until the analysis of dengue virus and genetic analysis are completed. These samples will be stored at the research laboratory at Monash University Malaysia. The research specific study data will be stored securely for 5 years, according to Monash University research storage data policies and procedures (please refer http://www.researchdata.monash.edu/).

#### Statistical analysis

In each wave of data collection, the occurrence of recent primary and recent secondary infections will be examined using the approach described by Lima et al. [38]. Specifically IgM + ve and IgG-ve is interpreted as a recent primary infection. IgM + ve and IgG + ve, or IgM-ve and IgG + ve is interpreted as a recent secondary infection. IgM-ve and IgG-ve is interpreted as no recent infection. The rate of infection, and the rate of recent primary and secondary infections, (separately) can thus be estimated. Using the panels of data, it will further be possible to examine the rate of misclassification, if temporal reversals between recent secondary and recent primary infections occur. Within the panels, logistic regression will be used to examine the binary relationship between the occurrence of primary and/or secondary infections and host socio-economic and demographic characteristics. The panel data will be formally analysed using a Generalized Estimating Equation (GEE) technique with a logit link function [39, 40]. Models will be developed for recent infections (both primary and secondary). The GEE approach estimates population averaged effects taking account of the correlated nature of repeated measurements in the panel data [40]. An autoregressive order 1 (AR1) correlation structure is assumed, but alternative correlation structures will also be tested. The association of socio-economic and demographic, pre-existing medical conditions, vaccination and family history covariates will also be examined.

## Discussion

Unplanned urbanization, increased population density and poor waste management in urban areas are playing a major role in the global spread of dengue. However, this is not the scenario in Malaysia where dengue is prevalent in both urban and rural areas [34]. In the current decade, dengue is considered to be one of the neglected tropical diseases (NTDs), and also an infectious disease of poverty (IDP) [41]. There is, however, a debate about whether dengue is really a disease of poverty, and there is a lack of empirical studies to inform this [42]. To reduce this knowledge gap, it becomes essential to conduct research across geographical settings and among study populations living under different socioeconomic conditions.

This prospective cohort study will facilitate the identification of the early phases of illness, and will examine the association of the clinical manifestations of the early phase with the entire course of illness. In particular, it will estimate the burden of asymptomatic dengue at the community level of Malaysia which remains as a latent source of further symptomatic dengue infection. This study will also identify a) which DENV serotypes are circulating both in the human hosts and mosquito vectors at the community level; b) how they interact with hosts’ immune status; and c) how socioeconomic and demographic conditions as well as pre-existing medical conditions of hosts contribute to the disease’s complexity. It will also examine the relationship between different virus serotypes and host genotypes.

To date, there is no effective vaccine or specific treatment for dengue [43]. The Malaysian government is interested in effectiveness and implementation studies before making the decision to start using the current vaccine [44], which is limited because of the difficulties developing a single vaccine for all four DENV serotypes. Our study may additionally contribute to research into the efficacy of the dengue vaccine for the Malaysian population by developing an understanding of the nature of both asymptomatic and symptomatic dengue infections and their associated factors related to the virus, hosts and vectors. The study will provide evidence for policy makers to develop tools for raising awareness among the general population about prevention and control measures. It will also support the development of systems to reduce the rate of morbidity and mortality, by initiating early identification of dengue cases and ensuring timely treatment at in-patient and out-patient facilities. This longitudinal study design may have some limitations. The SEACO census found that younger participants migrate out of the study area either for education or career purpose [33]. This may affect the retention rate.

## Abbreviations

A&E, Accident & Emergency; AR1, autoregressive order 1; CEC, Community Engagement Committee; DALY, disability-adjusted life year; DENV, dengue virus; EDTA, Ethylenediaminetetraacetic acid; ELISA, enzyme-linked immunosorbent assay; FcγR, Fc-gamma receptor; GEE, Generalized Estimating Equation; GPs, General Practitioners; HCT, haematocrit; HDSS, health and demographic surveillance site; HLA, human leukocyte antigen; IgG, Immunoglobulin G; IgM, Immunoglobulin M; IDoP, Infectious disease of poverty; JAK1, Janus Kinase1; KK, Klinik Kesihatan; mls, millilitres; MOH, Ministry of Health; NGS, Next Generation Sequencing; NS1, nonstructural protein 1; NTDs, Neglected Tropical Diseaes; ODK, Open Data Kit; PCR, polymerase chain reaction; PRNT, Plaque Reduction Neutralization Test; SEACO, South East Asia Community Observatory; SMS, short message service; SNPs, single nucleotide polymorphisms; WHO, World Health Organization

## Additional file

Additional file 1: Multilingual abstracts in the six official working languages of the United Nations. (PDF 302 kb)
