# Supplementary material for: A community-based prospective cohort study of dengue viral infection in Malaysia: the study protocol
Source: Infect Dis Poverty. 2016 Aug 11;5:76. doi: 10.1186/s40249-016-0172-3 (PMC4980774; doi:10.1186/s40249-016-0172-3)
Supplement: Additional file 1: — Multilingual abstracts in the six official working languages of the United Nations. (PDF 302 kb) [file 40249_2016_172_MOESM1_ESM.pdf]

Translation of the abstract into the six official working languages of the United Nations

دراسة مجتمعية للأتراب المحتملين لعدوى حمى الضنك الفيروسية في ماليزيا: بروتوكول دراسة

نوروزي قمر جهان، مختار بونجوت أحمد، والدكتور أمريتا داناو، تشيونج يويت منج، لاو وي مينغ، دانيال د. ريدباث، باسكال ألوتي، أنور زيني، مود الفيرا فيبس، كويك كيا فات، أمان بن ربه، روذير سراج الدين، أحمد عبد الباسط أحمد فاتان ، فيصل عدلي الغفار، حمدان بن أحمد، إحسان عثمان، شريفة سيد حسن

ملخص

خلفية: على الصعيد العالمي، تشكل التهابات الضنك عبئا كبيرا على الصحة العامة . في العقود الأخيرة، أصبح الضنك شديد التوطن في ماليزيا حيث تتواجد الأنماط المصلية الأربعة لفيروس حمى الضنك. والهيمنة الدورية للأنواع الفرعية تساهم في وجود نمط من الفاشيات الكبرى. العواقب يمكن ملاحظتها في حالات ارتفاع حالات حمى الضنك المبلغ عنها والوفيات الناجمة عن حمى الضنك . فهم التفاعل المعقد لفيروس حمى الضنك، العائل البشري والبعوض الناقل على مستوى المجتمع المحلي قد يساعد في وضع استراتيجيات لمعالجة هذه المشكلة.

الطرق: سيتم إجراء دراسة على الأتراب المحتملين في منطقة سيجامات في ولاية جوهور في شبه جزيرة ماليزيا. حصل الباحثون على موافقة من اللجنة الماليزية للأخلاقيات البحوث الطبية ولجنة جامعة موناش لأخلاقيات البحوث على البشر . وستجرى الدراسة في موقع في ماليزيا للصحة والمراقبة الديموغرافية خلال فترة سنة واحدة في ثلاث مناطق مختلفة في طبيعتها الديموغرافية (في المناطق الحضرية وشبه الحضرية والريفية). تشمل الدراسة البالغين الأصحاء (الذكور والإناث) الذين تتراوح أعمارهم بين 18 سنة وما فوق، من ثلاث مجموعات عرقية (الملايو والصينية والهندية). حجم العينة تم حسابها باستخدام طريقة فليس مع تصحيح الاستمرارية333.سيتم إجراء استطلاع مراقبة الإصابة للمشاركين لتحديد الحالات التي لا تبدو عليها أعراض، والحالات الصحية خلاف ذلك؛ والحالات المصابة بحمى الضنك التي يتم التعامل معها كمرضى خارجيين . والحالات المصابة بحمى الضنك المدخلة إلى المستشفى. وسيتم إجراء تحليل جيني للمشاركين لتحديد ما إذا كانت هناك علاقة بين الاستعداد الوراثي وشدة المرض. كما سيتم الرجوع للتاريخ الطبي المفصل لكل حالة، والإصابات السابقة بعدوى حمى الضنك، وتاريخ التطعيم ضد الفيروسات المصفرةال أخرى مثل التهاب الدماغ الياباني والحمى الصفراء، والتاريخ العائلي للإصابة بحمى الضنك. وبالإضافة إلى ذلك، ستتم مراقبة البعوض في وقت واحد في مناطق إجراء البحث لتحديد التصنيف الجزيئي للناقلات.

المناقشة: ستقدر نتائج البحث عبء حمى الضنك عديمة الأعراض وذات أعراض على مستوى المجتمع المحلي . وستدرس أيضا العلاقة بين الأنماط المصلية للفيروس ومورثات العائل، وجمع المظاهر السريرية للمرحلة مبكرة مع كامل مراحل المرض.

Translated from English version into Arabic by Mahmoud Sami, through

TRANSLATORS
WITHOUT BORDERS

在马来西亚以社区为基础的登革病毒感染前瞻性队列研究：研究计划

Nowrozy Kamar Jahan, Mohtar Pungut Ahmad, Dr. Amreeta Dhanoa, Cheong Yuet Meng, Lau Wee Ming, Daniel D Reidpath, Pascale Allotey, Anuar Zaini, Maude Elvira Phipps, Quek Kia Fatt, Aman Bin Rabu, Rowther Sirajudeen, Ahmad AbdulBasitz Ahmad Fatan, Faidzal Adlee Ghafar, Hamdan Bin Ahmad, Iekhsan Othman, Sharifah SyedHassan

摘要：

引言：在全球范围内，登革热构成重大的公共卫生负担。近年来，随着 4 种不同血清型的传播，马来西亚已成为登革热的高流行区。各亚型周期性的流行构成了该疾病的主要爆发模式。由此造成的结果就是登革热发病率和相关死亡病例数的上升。在社区水平了解登革病毒与宿主和媒介按蚊之间的复杂的相互关系也许能够帮助我们制定更好的策略来解决这一难题。

方法：在马来西亚柔佛州昔加末区开展前瞻性队列研究，本研究获得马来西亚医学研究伦理委员会和莫纳什大学人类研究伦理委员会的批准。本研究将基于马来西亚健康和人口监测点在 3 种不同的环境下（城市、城乡结合部、乡村）开展为期 1 年的工作。本研究将从 3 个族群（马来人、中国人和印度人）中招募 18 岁以上的健康成年男女。通过连续性校正弗莱斯方法计算样本量为 333。通过对参与血清监测的受试者检测来确认无症状者及健康者，感染者被作为门诊病人来管理，并入院治疗。对受试者进行遗传分析来确认遗传因素与疾病程度之间是否存在一定的关系。本研究收集详细的病史，既往登革热感染情况，

包括日本脑炎和黄热病等其他黄热病毒疫苗的接种史，以及登革热感染的家族史资料。此外，还在招募地区开展媒介蚊虫的监测并进行分子鉴定。

讨论：本研究将在社区水平评估无症状和有症状登革病毒感染的疾病负担。同时，本研究还将检测病毒型和宿主基因型之间的关系，及早期临床表现和整个病程之间的关系。

Translated from English version into Chinese by Feng Xin-Yu, edited by Yang Pin, through

CHINESE CENTER FOR DISEASE CONTROL AND PREVENTION
NATIONAL INSTITUTE OF PARASITIC DISEASES

Une étude de cohorte communautaire sur la contamination par le virus de la dengue en Malaisie : protocole d’étude

Nowrozy Kamar Jahan, Mohtar Pungut Ahmad, Dr. Amreeta Dhanoa, Cheong Yuet Meng, Lau Wee Ming, Daniel D Reidpath, Pascale Allotey, Anuar Zaini, Maude Elvira Phipps, Quek Kia Fatt, Aman Bin Rabu, Rowther Sirajudeen, Ahmad AbdulBasitz Ahmad Fatan, Faidzal Adlee Ghafar, Hamdan Bin Ahmad, Iekhsan Othman, Sharifah SyedHassan

Résumé

Contexte: La dengue est un important problème de santé publique à l'échelle mondiale. Ces dernières décennies, la Malaisie est devenue un pays où la dengue est hyper-endémique et où circulent simultanément les quatre sérotypes du virus. La dominance cyclique de sous-types contribue à un schéma de flambées majeures. Les conséquences peuvent être observées à travers l’incidence accrue des cas déclarés de dengue et des décès liés au virus. Comprendre l’interaction complexe du virus de la dengue, ses hôtes humains et les moustiques vecteurs de la maladie à l’échelle de la communauté pourra peut-être nous aider à développer des stratégies pour traiter le problème.

Méthodes: Une étude de cohorte prospective sera menée dans le district de Segamat, dans l’état de Johor, au sein de la péninsule malaisienne. Les chercheurs ont reçu l’autorisation du Malaysian Medical Research Ethics Committee (Comité d’éthique pour la recherche médicale en Malaisie) et du Monash University Human Research Ethics Committee (Comité d’éthique pour la recherche sur les êtres humains de l’Université de Monash). L’étude sera menée sur un site de surveillance sanitaire et démographique malaisien sur une période d’un an et dans trois milieux différents (urbain, semi-urbain et rural). Elle recrutera des adultes (hommes et femmes) en bonne santé âgés de 18 ans et plus et issus de trois groupes ethniques différents (malais, chinois et indien). La taille de l’échantillon, calculé selon la méthode de Fleiss avec une correction de continuité, est de 333. L’analyse du sérum des participants servira à identifier les cas asymptomatiques ou en bonne santé, les cas de dengue traités en tant que patients externes et les cas de dengue hospitalisés. Une analyse génétique des participants sera menée afin de déterminer s’il existe un rapport entre une prédisposition génétique et la gravité de la maladie. Les antécédents médicaux détaillés, les antécédents de dengue, de vaccination contre d’autres flavivirus comme l’encéphalite japonaise ou la fièvre jaune ainsi que les antécédents familiaux d’infection par la dengue seront également recueillis. En parallèle, une analyse des moustiques sera menée sur les zones de recrutement afin de déterminer la taxonomie moléculaire des vecteurs en circulation.

Discussion: Les résultats de la recherche permettront d’estimer l’enjeu que représente la dengue symptomatique et asymptomatique à l’échelle de la communauté. Elle étudiera également le rapport entre les sérotypes du virus et ceux de l’hôte ainsi quet le lien entre la manifestation clinique de la phase précoce et le déroulement complet de la maladie.

Translated from English version into French by Carine Toucand, through

TRANSLATORS
WITHOUT BORDERS

Перспективное когортное исследование вирусной инфекции денге на уровне общины в Малайзии: протокол исследования

Наврози Камар Джахан, Мохтар Пунгут Ахмад, д-р. Амрита Дханоа, Cheong Yuet Meng, Lau Wee Ming, Дэниел Д. Рэйдпат, Паскаль Аллотэ, Ануар Зайни, Мод Элвира Фиппс, Куэк Киа Фэтт, Аман Бин Рабу, Роутер Сираджудин, Ахмад АбдулБазитц Ахмад Фатан, Файдзал Адли Гафар, Хамдан Бин Ахмад, Иексан Отман, Шарифа СиедХассан

Реферат

Исходная информация: В глобальном масштабе инфекционное заболевание денге представляет значительное бремя для общественного здравоохранения. В последние десятилетия в Малайзии наблюдаются гиперэндемические масштабы распространения денге с параллельной циркуляцией четырех серотипов вируса денге. Циклическое преобладание подтипов влияет на характер эпидемических вспышек. Последствия этого наблюдаются в росте зарегистрированных случаев заболевания денге и связанных с ними смертельных исходов. Понимание комплексного взаимодействия между вирусом денге, его человеческим организмом-носителем и москитами-переносчиками вируса на уровне общины может содействовать выработке стратегий решения этой проблемы.

Методы: Перспективное когортное исследование будет проводиться на территории Полуостровной Малайзии (Peninsular Malaysia) в районе Сегамат (Segamat) штата Джохор (Johor State). Исследователи получили одобрение Комитета по этике медицинский исследований Малайзии (Malaysian Medical Research Ethics Committee) и Комитета по этике исследований на человеке университета Монаш (Monash University Human Research Ethics Committee). Исследование будет проводиться в течение одного года на расположенном в Малайзии участке для наблюдения за состоянием здоровья и демографией населения в трех различных средах (городской, полугородской и сельской). В исследовании примут участие здоровые представители (женского и мужского пола) трех этнических групп (малайцы, китайцы и индийцы) в возрасте 18 лет и старше. Объем выборки рассчитан по методу Флейсса с поправкой на непрерывность 333. Будет проводиться серологический эпиднадзор для идентификации бессимптомных и здоровых в других отношениях случаев, а также случаев амбулаторного лечения больных лихорадкой денге и госпитализированных больных лихорадкой денге. Для установления наличия каких-либо взаимосвязей между генетической предрасположенностью и тяжестью болезни будет проведен генетический анализ участников. Также будут собраны подробные медицинские карты участников, информация о прошлых инфекционных заболеваниях денге, прививках против других флавивирусов, как например, японского энцефалита или желтой лихорадки; а также информация о случаях заражения вирусом денге среди членов семьи. Кроме того, в местах проживания участников исследования одновременно будет проводиться наблюдение за москитами в целях определения молекулярной классификации циркулирующих переносчиков.

Обсуждение: Данные, полученные в ходе исследования, помогут оценить бремя бессимптомной и симптоматической лихорадки денге на уровне общины. Кроме того, будет рассмотрена взаимосвязь между серотипами вируса и генотипами организма-носителя, а также связь между клиническим проявлением на ранней стадии и полным курсом течения болезни.

Translated from English version into Russian by Tatyana Johnson, through

TRANSLATORS
WITHOUT BORDERS

Estudio prospectivo de grupo basado en la comunidad de la infección viral de dengue en Malasia: protocolo del estudio

Nowrozy Kamar Jahan, Mohtar Pungut Ahmad, Dr. Amreeta Dhanoa, Cheong Yuet Meng, Lau Wee Ming, Daniel D Reidpath, Pascale Allotey, Anuar Zaini, Maude Elvira Phipps, Quek Kia Fatt, Aman Bin Rabu, Rowther Sirajudeen, Ahmad AbdulBasitz Ahmad Fatan, Faidzal Adlee Ghafar, Hamdan Bin Ahmad, Iekhsan Othman y Sharifah SyedHassan

Resumen

Información de referencia: Las infecciones por dengue constituyen una importante carga para la salud pública en a escala mundial. Durante las pasadas décadas, Malasia se ha convertido en un país hiperendémico de dengue por la presencia simultánea de los cuatro serotipos del virus del dengue. El dominio cíclico de los subtipos facilita que se produzcan importantes brotes de la enfermedad. Las consecuencias se pueden observar en la creciente incidencia de los casos conocidos de dengue y en muertes relacionadas con el dengue. Conocer la compleja interacción del virus del dengue, sus huéspedes humanos y los mosquitos como vectores en las comunidades puede ayudar a desarrollar estrategias para afrontar el problema.

Métodos: Se llevará a cabo un estudio prospectivo de grupo en el distrito de Segamat, en el estado de Johor situado en la Malasia peninsular. Los investigadores han recibido el visto bueno del Comité Ético de Investigación Médica de Malasia y del Comité de Ética de Investigación Humana de la Universidad de Monash. El estudio será realizado en un centro de vigilancia sanitaria y demográfica de Malasia a lo largo de un año en tres tipos de emplazamientos (urbano, semiurbano y rural). El estudio se realizará sobre adultos sanos (hombres y mujeres) de 18 años o más, pertenecientes a tres grupos étnicos (malayo, chino e indio). El tamaño de la muestra calculado mediante el método de Fleiss con corrección de continuidad es 333. Se llevará a cabo la serovigilancia de los participantes para identificar los casos asintomáticos, a menos que se trate de individuos sanos; casos con fiebre por dengue gestionados como pacientes ambulatorios; y casos con fiebre por dengue de individuos que han ingresado en un hospital. Se llevará cabo un análisis genético de los participantes para determinar si existe una relación entre la predisposición genética y la gravedad de la enfermedad. También se recogerá un historial médico detallado, el historial pasado de infección por dengue, historial de vacunación frente a otros flavivirus como la encefalitis japonesa y la fiebre amarilla, así como el historial familiar de infección por dengue. Además se realizará simultáneamente una vigilancia de los mosquitos en las áreas estudiadas con el fin de determinar la taxonomía molecular de los vectores circulantes.

Conclusión: Las averiguaciones de la investigación permitirán obtener una estimación de la incidencia del dengue asintomático y sintomático al nivel de la comunidad. También examinará la relación entre los serotipos del virus y los genotipos del huésped, así como la correspondencia entre la manifestación clínica de la fase inicial y la evolución de la enfermedad.

Translated from English version into Spanish by SergioLorenzi, through

TRANSLATORS
WITHOUT BORDERS
